# Renal artery pseudoaneurysm following robot assisted nephron sparing surgery: two case reports

**DOI:** 10.1186/s13256-024-04510-6

**Published:** 2024-04-19

**Authors:** Ravi Mohan Mavuduru, Shiraz Akif Mohd Ziauddin, Girdhar Singh Bora, Ujjwal Gorsi

**Affiliations:** 1 Department of Urology, PGIMER, Chandigarh, India; 2 Department of Urology, AIIMS, Nagpur, 160012 India; 3 Department of Radiodiagnosis, PGIMER, Chandigarh, India

**Keywords:** Pseudoaneurysm, Angioembolization, Nephron sparing surgery, Digital subtraction angiography

## Abstract

**Background:**

Renal artery pseudoaneurysm following partial nephrectomy is a rare entity, the incidence of this entity is more common following penetrating abdominal injuries, percutaneous renal interventions such as percutaneous nephrostomy(PCN) or Percutaneous nephrolithotomy (PCNL). Although rare, renal artery pseudoaneurysm can be life threatening if not managed timely, they usually present within two weeks postoperatively with usual presenting complains being gross haematuria, flank pain and/or anaemia.

**Case presentation:**

We report case of two female patients 34 and 57 year old respectively of South Asian ethnicity, presenting with renal artery pseudoaneurysm following left sided robot assisted nephron sparing surgery for interpolar masses presenting clinically with total, painless, gross haematuria with clots within fifteen days postoperatively and their successful treatment by digital subtraction angiography and coil embolization.

**Conclusion:**

Renal artery aneurysm is a rare fatal complication of minimally invasive nephron sparing surgery however considering the preoperative and intraoperative risk factors for its development and prompt suspicion at the outset can be life saving with coil embolization of the bleeding arterial aneurysm.

## Background

Partial nephrectomy open, laparoscopic or robotic is the current standard of surgical treatment of small renal mass cT1 stage. Minimally invasive surgery combined with advanced surgical skills has made the latter two techniques quite popular with added advantage of preventing long term morbidity of renal insufficiency associated with radical nephrectomy. One of the postoperative complications following partial nephrectomy is bleeding either into the pelvicalyceal system presenting as gross haematuria or in the pararenal space as perinephric haematoma manifesting as flank pain, however delayed bleeding secondary to arterio-venous fistula and pseudoaneurysm formation is a rare phenomenon with an incidence of 2% in minimally invasive surgery compared to 1% in open partial nephrectomy according to a study [1]. Usually these patients present clinically with one of the classical triads within fifteen days postoperatively although few cases of delayed presentation after 2- 4 months have also been described in the literature [2] also asymptomatic cases have also been reported. Renal angiography and selective bleeding artery embolization under fluoroscopic control is a safe and effective intervention with minimal morbidity and maximal nephron sparing. Herein we report two cases of the RANSS (Robot assisted nephron sparing surgery) operated at our institute with post operative complication of renal artery pseudoaneurysm, its successful management by intervention radiology and retrospective audit of the recorded surgical videos with the lessons learnt to avoid this fatal complication in future surgery. This case has been reported in line with the CARE guidelines [3].

## Case presentation

### Case 1

**Fig. 1:**
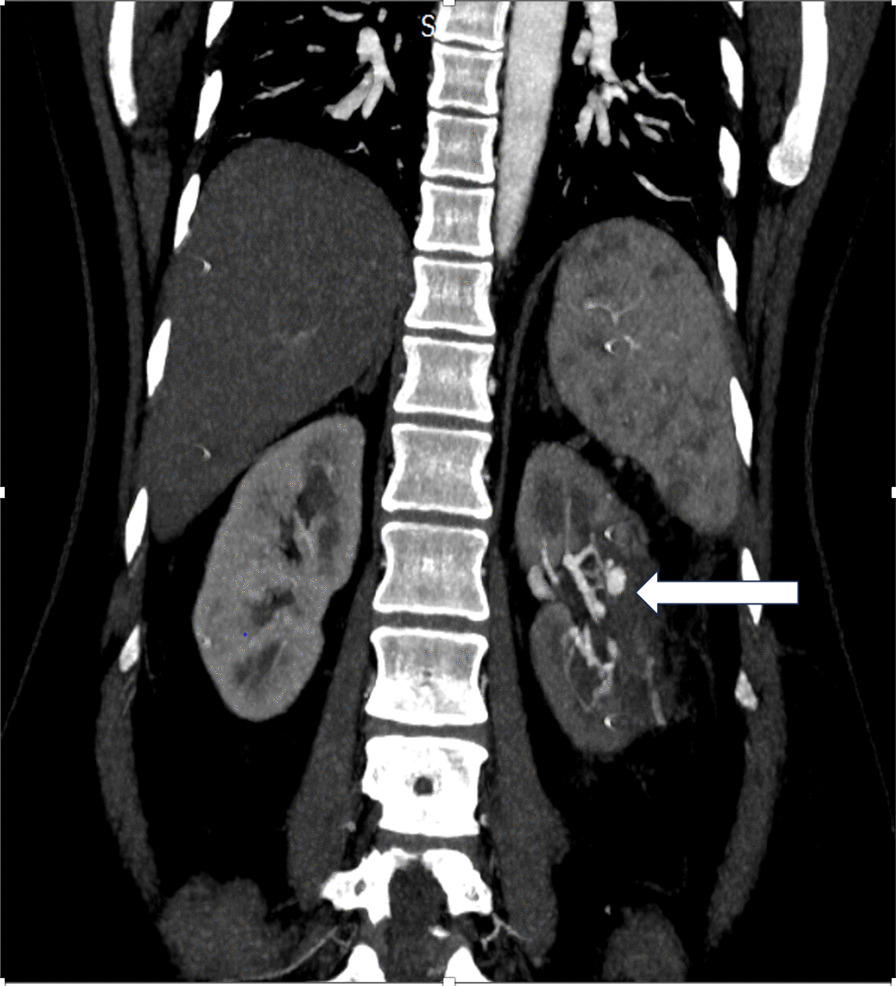
Pseudoaneurysm of the left interpolar branch of the posterior division of left renal Artery (white arrow)

**Fig. 2:**
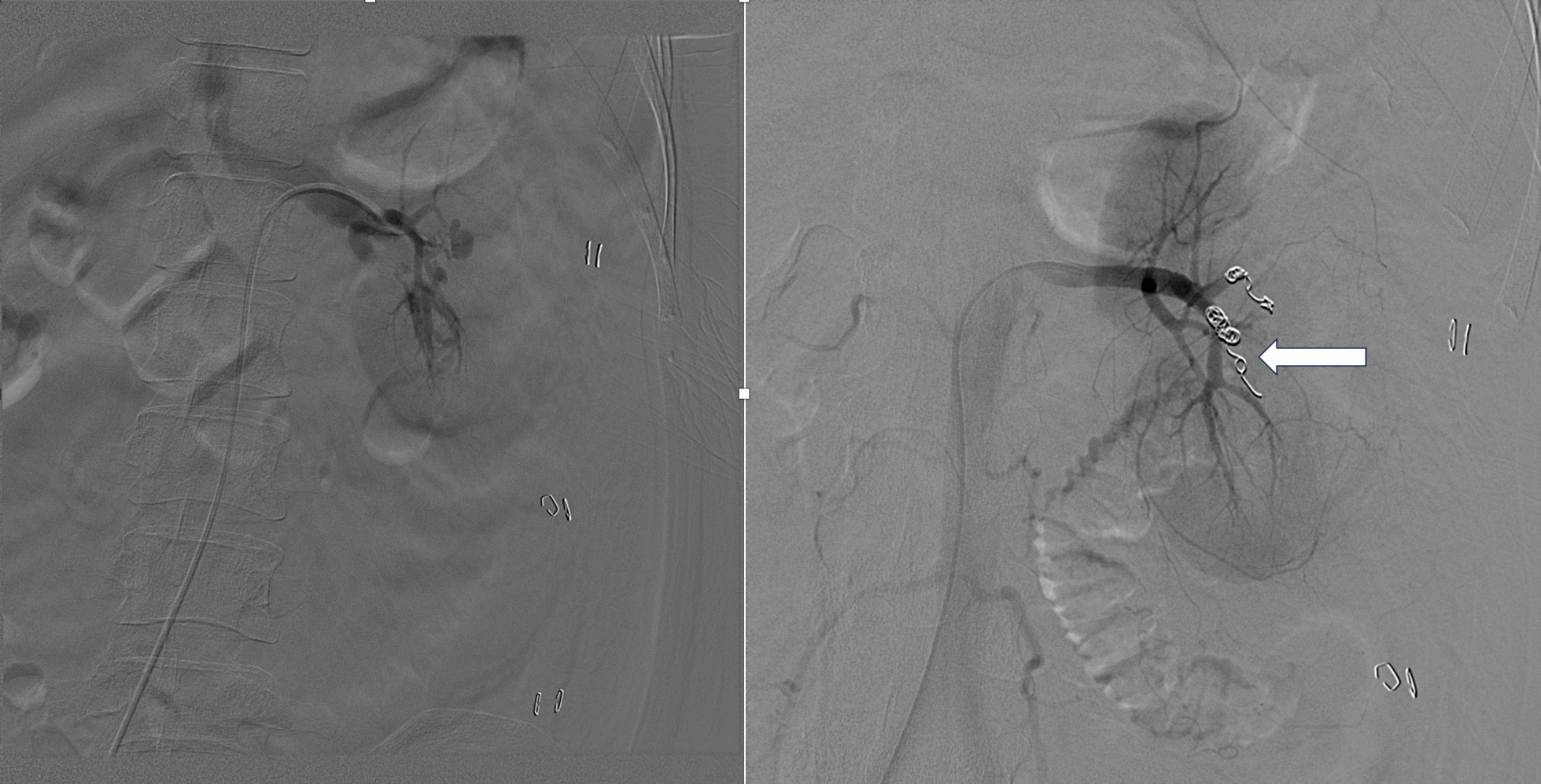
Coil embolization (white arrow) under digital subtraction angiography control for both pseudoaneurysm and arteriovenous fistula and their resolution

A 34 year old lady of South Asian ethnicity presented with incidentally detected left hilar 4 × 4 cm heterogeneously enhancing renal mass (cT1aN0M0) on CECT (Contrast-Enhanced Computed Tomography) with RENAL Nephrometry score of 7 a. After giving written informed consent the patient underwent uneventful robot assisted nephron sparing surgery, the total operative time was 2.30 h, intraoperative blood loss was 300 ml and warm ischemia time was 30 min, eneucleoresection of the tumour was done. The postoperative period was uneventful and patient was discharged without any complications on fifth post operative day. Histopathology was suggestive of renal cell carcinoma chromophobe type. She presented in emergency on fourteenth postoperative day, with complain of gross, total, painless haematuria with clots since one day with slight tachycardia of 92/min but in a haemodynamically stable state. She underwent CECT urography with renal angiography showing the presence of mildly prominent left intersegmental arteries with a pseudoaneurysm of the segmental artery and a small arteriovenous fistula (AVF) of size 6.6 × 5.6 mm arising from interpolar branch of the posterior division of left renal artery (Fig. 1). Coil embolization under digital subtraction angiography (DSA) control was done for both pseudoaneurysm and AVF (Fig. 2) and patient was discharged post procedure after brief observation in stable condition. Follow up CECT abdomen at 6 and 12 months showed no residual pseudoaneurysm or tumour recurrence. The serum creatinine levels were within normal limit and the post SAE renal function on Tc99 dynamic renal scintigraphy scan was 32% with preserved cortical function at 1 year follow up.

### Case 2

**Fig. 3:**
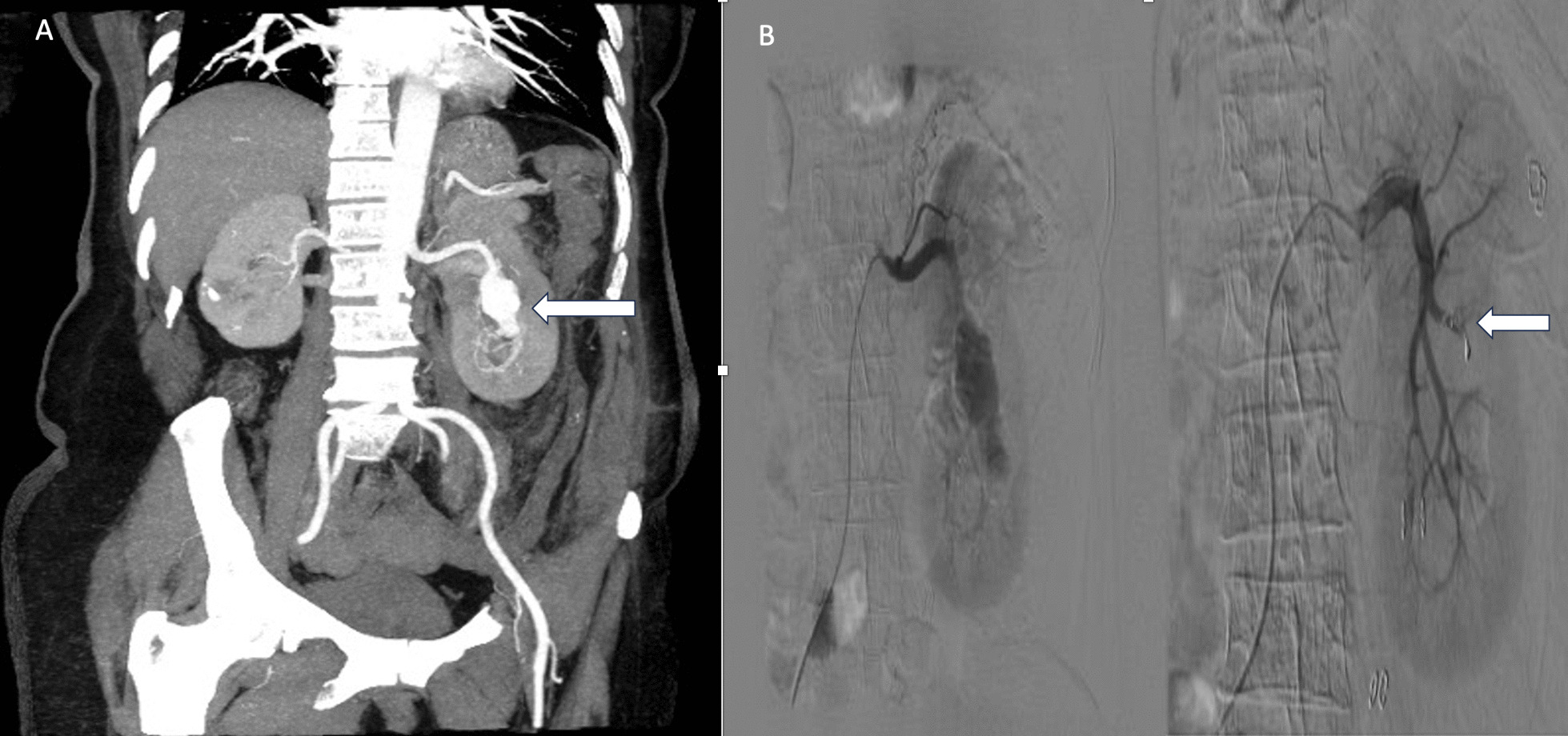
**A** Large pseudoaneurysm of size 4.6 x 1.9 x 2.5cm (white arrow), **B** Super selective coil angioembolization under DSA control (white arrow) with its resolution

A 57 year old lady of South Asian ethnicity underwent RANSS for an incidentally detected partially exophytic 5 × 5 cm interpolar left renal mass (cT1bN0M0), the RENAL nephrometry score was 7p, operative time was 2 h, 200 ml of intraoperative blood loss and warm ischemia time (WIT) of 35 min, eneucleoresection of the tumour was done. The perioperative period was uneventful and she was discharged in stable condition on fourth postoperative day. Histopathology was suggestive of clear cell renal cell carcinoma. She presented in emergency on fourteenth postop day with complain of left flank pain and gross, total, painless haematuria with slight tachycardia of 96/min but in a haemodynamic stable condition; the patient underwent CECT urography with renal angiography showing a large pseudoaneurysm of size 4.6 × 1.9 × 2.5 cm projecting into the PCS which was super selectively coil angioembolized under DSA control (Fig. 3A, B). The patient was discharged post procedure after brief observation in stable condition. Follow up CECT abdomen at 6 and 12 month showed no residual pseudoaneurysm or tumour recurrence. The serum creatinine levels were within normal limit and the post SAE renal function on Tc^99^ dynamic renal scintigraphy scan was 38% with preserved cortical function at 1 year follow up.

## Discussion

Nephron sparing surgery- open or minimally invasive is the contemporary state of art for the surgical management of small renal masses providing the benefit of nephron preservation which has a protective effect from landing into downhill renal insufficiency in the long run [4, 5]. There is an increasing application of MIPN (Minimally invasive partial nephrectomy) in more complex renal masses but at the cost of increased number of complications in recent times [6]. Intraoperative bleeding or delayed haemorrhage caused by either pseudoaneurysm or arteriovenous fistula formation remains most common complication of the above technique. Various management options for renal artery pseudoaneurysm includes conservative management in haemodynamically stable patients [7], angioembolization of pseudoaneurysm in unstable patients and nephrectomy in failed angioembolization cases. Renal artery pseudoaneurysm after partial nephrectomy as was first reported by Rezvani *et al.* in 1973 [8]. It can be a potentially life threatening complication, the proposed mechanism of formation of which is partial transection of the artery during tumour resection leading to bleeding into a contained perinephric hematoma, more so at the apex of the eneucleo wedge resection or during the suturing of the resected tumour bed when the underlying blood vessel is partially traumatized causing a false puncture in its wall [9]. Intraoperatively the risk factors for pseudoaneurysm formation includes the longer operative time and ischemia time, endophytic and posterior located tumours, higher RENAL nephrometry score, renal sinus exposure and operative blood loss of > 250 ml [10]. As in our case both the tumours were complex interpolar and both had pelvicalyceal system breach during the tumour resection with longer warm ischemia time. Selective renal artery angioembolisation (SAE) is an intervention radiological technique with high success rate of controlling postoperative haemorrhage from the pseudoaneurysm with maximal functional renal parenchymal preservation, contrary to the surgical approach for the same which can lead to almost always a total loss of the index renal unit when explored. The timing of the these clinically significant bleeding complications is usually within two weeks postoperatively [10] similar to our case. We propose various possible reasons of pseudoaneurysm formation which are as follows: 1. Tight running suture placement during haemostasis for vessel transection at the surgical bed, 2. Unnecessary parenchymal needle punctures during haemostasis, 3. Not checking the haemostasis after pneumoperitoneum deflation at the end of surgery to expose any impending bleeders. In our cases the post SAE renal function declined slightly in both the patients however the global renal function was within the normal limit as in line with Ghoniem *et al.* [11] where in 15 patients post SAE for bleeding renal artery pseudoaneurysm showed good renal function except for 1 solitary functioning kidney with a decline in post angioembolization. The loss in renal function can be due to either the loss of normal renal parenchyma (Penumbra) due to the end arterial nature of blood supply in kidney or the effect of nephrotoxic contrast media used during the angioembolization process on the postoperative ischemia recovering kidney. With the evolution of the surgical acumen during his career and application of this minimally invasive surgery to more clinically complex renal masses as the surgeon gains experience along with strict postoperative surveillance for the complications, there will be an increasing understanding and future prevention of this lethal complication as the operating surgeon improves upon the technical surgical expertise.

## Conclusion

- Renal artery pseudoaneurysm is a rare fatal complication of minimally invasive partial nephrectomy, risk factors being complex masses, longer operative and ischemia time, endophytic and posteriorly located tumours, PCS breech and renal sinus fat exposure during resection.
- SAE is an effective low morbidity radiological modality with maximal renal parenchymal and functional preservation after partial nephrectomy.
- Active surveillance and technical surgical revaluation by the surgeon improves the complication rates and allows the surgeon for application of his  improved technique to more complex renal mass lesions confidently in his surgical learning curve.

## Data Availability

Not applicable.
